# Spark plasma welding joining of copper- AISI4140 steel: Microstructures and mechanical properties

**DOI:** 10.1016/j.heliyon.2023.e21364

**Published:** 2023-10-23

**Authors:** Mehdi Naderi, Mohammad Reza Toroghinejad, Ahmad Kermanpur

**Affiliations:** Department of Materials Engineering, Isfahan University of Technology, Isfahan, 84156-83111, Iran

**Keywords:** Spark plasma welding, Copper- AISI4140 steel joint, Diffusion affected zone, Unjointed areas, Micropores, Joining strength

## Abstract

The main aim of this research study was to characterize the microstructures and mechanical properties of Cu-AISI4140 steel solid-state joining created via the spark plasma welding (SPW) method with and without using molds. To explore the effect of mold on the joining of copper/steel, the SPW process was done at 650 °C for 30 min under the pressure of 20 MPa, with and without mold. Microstructural evaluations indicated the diffusion-affected zone (DAZ) in the SPW process increased with mold, as compared to the sample considered in the absence of the mold process. Also, the SPW process with the mold, in response to the lack of the formation of the oxide layer and dead zone, was affected by the process pressure, in comparison to that without the mold process, leading to the reduction of unjointed areas and the formation of micropores constrained at the joining interface; as such, the joining strength was increased from 42 MPa to 90 MPa. The elevation of the applied pressure from 20 MPa to 40 MPa at 650 °C resulted in enhancement of the joining strength up to 106 MPa, but it had no perceptible effect on raising the strength and diffusion affected zone (DAZ) of the joint.

## 1 Introduction

Copper and steel are among the most practical metals in different industries [[1], [2], [3], [4], [5], [6], [7]]. With the development of contemporary industries, materials with unique properties are progressively in demand. Nevertheless, a metal or alloy has limitations in terms of costs or presentable properties; also, in some special practical conditions, it cannot fulfil all requirements. Thus, in recent years, special attention has been paid to bimetallic composites, which are composed of dissimilar metals worldwide. Bimetallic composites, which belong to advanced materials technology, are prepared from two types of metal with different physical, chemical and mechanical properties, at whose interface there is a metallurgic bond. Copper-steel bimetallic composites can present a combination of conductivity and strength effectively. Thus, a copper-steel composite is practical in power transmission and generation, heat transfer components, aeronautics, automobile, cooling, electric, and electronic industries [8].

The weldability of dissimilar metals is determined by atomic radius, crystalline structure, and the components' solubility in a solid and molten state [[9], [10], [11]]. Considering the difference in the chemical composition and some thermophysical and thermodynamic properties of copper and iron, such as the difference in thermal conductivity and thermal expansion coefficient and the limited solubility of copper and iron, welding and joining steel and copper can be challenging [12,13]. The problems related to the arc welding of copper and steel can be rectified using compatible filler materials and their precise selection. Accordingly, the use of another metal that is soluble with each of the dissimilar metals is required for establishing the joint [14]. Reports have shown that the presence of nickel in filler materials is very effective in achieving high strength [[14], [15], [16], [17]]. Nevertheless, considering research done on copper-steel joints via arc welding processes, due to the high heat conductivity of copper, the heat generated at the joining site fades away rapidly, and achieving the copper melting temperature along the molten welding process will be difficult. Of course, to solve these problems, processes such as grooving, preheating and multi-pass welding have been used [[18], [19], [20], [21], [22], [23], [24], [25]]. Meanwhile, copper and steel have low solubility thresholds. Thus, achieving a joint with desirable mechanical properties via the molten welding process is very challenging, whereby the formation of the sediments created during melting and freezing would cause aggravation of the joint's corrosion [26,27]. Also, due to the high input heat generated when using different methods of arc welding, in the heat affected zone (HAZ) of copper and copper alloys, grain growth and decline of mechanical properties can occur; in tensile tests, fracture occurs from this zone, and the final strength is lower than that of the copper-base metal [18,28]. However, in the research conducted by Oliveira et al. [29], due to the precipitation of the alpha phase and the increase of strength in the fusion zone and HAZ, failure did not occur in these areas. Welding processes in the solid state can resolve the problems related to arc welding to some extent, providing the possibility of achieving a suitable joining strength [30]. Of course, there are limitations in carrying out the process and the dimensions and geometry of the parts. In the research done by Deregtelo et al. [27], copper and stainless steel sheets were joined through explosive welding. Under optimal process conditions, due to the formation of waves at the interface and the increased contact area of the joint, no shear was seen in the interface after the shear-tensile test. Kimura et al. [31] also showed that friction welding of copper steel provided higher tensile strength, as compared to the base metal. Also, Lee et al. [32] welded copper alloy and STS329 without using an interlayer at 890 °C, under the pressure of 7 MPa for 1 h.

In the conventional methods of solid-state welding, establishing diffusion bonds requires processes at relatively high temperatures or a long time. Pulsed electrical current bonding, which is also called spark plasma welding (SPW), is a process by which the joining between materials is created through applying an electric current. By applying the pulsed DC current, plasma is formed at the interface of the contact areas of the pieces [33]. Thus, through the concurrent application of pressure and pulsed current, atomic diffusion occurs at the interface, leading to the possibility of joining different materials within a shorter time [30].

The main parameters of the SPW process include temperature, pressure, time, and electric current. Over the last three decades, relatively extensive research has been done to examine the effect of SPW process parameters on the joining of different materials. In the research done by Shen et al. [34], with the elevation of the process temperature up to 1100 °C, the joining of high-speed steel with strength close to the base metal was achieved. With further temperature elevation, the desired strength was obtained within a shorter time. According to results obtained by Goxy et al. [35], the thickness of the diffused region was increased by elevating the process temperature. In another research study by Nakao et al. [30], as the process temperature of joining the pure copper-AISI304 stainless steel was increased, the joining strength was raised too. The highest extent of joining strength was achieved at 700 °C under the pressure of 20 MPa, with failure occurring in the pure copper.

Based on the results of studies, in the Spark Plasma Sintering (SPS) process, the elevation of the process pressure would lead to a reduction of the temperature [36]. Augmentation of temperature and pressure would lead to an enlarged contact area in the initial stages and hence, the increased atomic diffusion at the joint's interface [37]. Based on the studies, atomic diffusion during the SPW process would also be affected by the electric current [38]. In Munir et al. [36], it was shown that applying current in the SPW process would elevate the vacancy concentration in copper and aluminum alloys. Cahill et al. [39] also showed that the pulsed DC current in the SPW process had a direct effect on the diffusion of Ca^2+^ and Sr^2+^ ions at the interface of the SrB_6_–CaB_6_ diffused pair. Further, Zhang et al. [40] compared the joining of chromium carbide (Cr_3_C_2_) and nickel by applying a current and without it. Based on the obtained results, the diffusion coefficient was increased with temperature elevation, showing a clear dependence on the current as well as its direction.

Although some research has been done on the joining of metals and various materials through the SPW process in the last three decades, there is still no research on the effect of the use of mold in the SPW process. Thus, this research evaluated microstructures and mechanical properties on the copper-AISI4140 steel joint via the SPW process at 650 °C for 30 min, under the pressure of 20 MPa, with and without mold.

## 2 Experimental

For the joining of copper-steel AISI4140 via the SPW process, pure copper with a purity of around 99.4 % and AISI4140 steel were prepared. Table 1 presents the results of the chemical analysis of steel AISI4140. Steel AISI4140, which is known by the commercial name Mo40 in the industry, is mostly used as quenched and tempered. Also, the microstructure of the quenched and tempered steel is finer than the equilibrium ones, which can affect the diffusion of copper into steel. Accordingly, the initial samples for the joining underwent quenching and tempering heat operations. The austenite temperature was considered 850 °C and austenite time was taken to be 30 min. All samples were quenched in oil. The surface hardness of the quenched steel was measured at 520 HV_10_. To perform the tempering process, the samples were exposed to 620 °C for 2 h and then air cooled. The hardness of the tempered steel was measured at 287HV_10_.

**Table 1:** Results of spark emission spectrometric (SES) analysis of steel AISI4140 used in this research (wt%).

	%C	%Si	%Mn	%Mo	%Cr
**Standard AISI**	0.38–0.43	0.15–0.30	0.75–1.0	0.15–0.25	0.80–1.10
**SES analysis**	0.41	0.26	0.8	0.17	1.15

To perform copper-steel joining processes via SPW, the initial samples of pure copper and steel, cylinders with a diameter of 20 mm and a height of 25 mm were considered. To remove surface contaminations, the samples were placed inside an acetone-containing ultrasonic bath for 10 min.

**Fig. 1:**
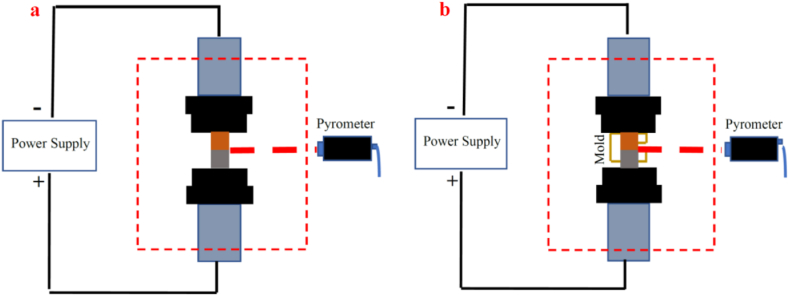
A schematic of the SPW process: a) without mold and b) with mold.

In this research, the SPW process was done using a SPS device (model of 10 KA-60 T, made In Iran). To explore the effect of mold, SPW processes were done with and without mold. Fig. 1 displays a schematic of the SPW process with (Fig. 1a) and without mold (Fig. 1b). In the mold-free process, the prepared surfaces of the samples were stacked on top of each other. The steel sample was placed on a graphite separator; after placing the samples, the graphite separator was placed on the pure copper sample. Next, the temperature measurement region was adjusted using a pyrometer at the center of the joint of the two samples, so that the joining site would be measured using a pyrometer along the process.

**Fig. 2:**
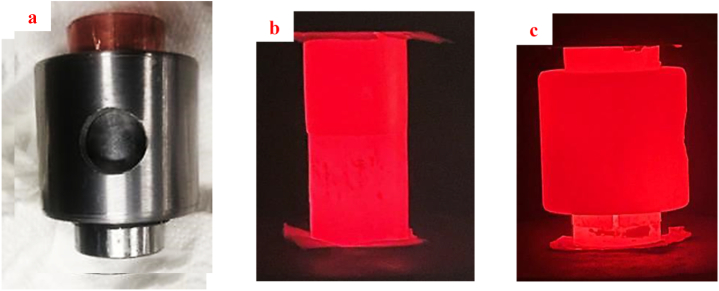
a) The metal mold used in the SPW process and the copper-steel jointed samples along the SPW process, b) without mold and c) with mold.

Fig. 2a indicates the metal mold used in this research. The metal mold was made of steel AISI4140; to perform the process, by placing the copper and steel sample inside the mold, the SPW process was done. To prevent the adhesion of the samples to the mold along the process, a graphite sheet was used. Fig. 2b and c show the copper-steel samples along the SPW process with and without mold.

**Fig. 3:**
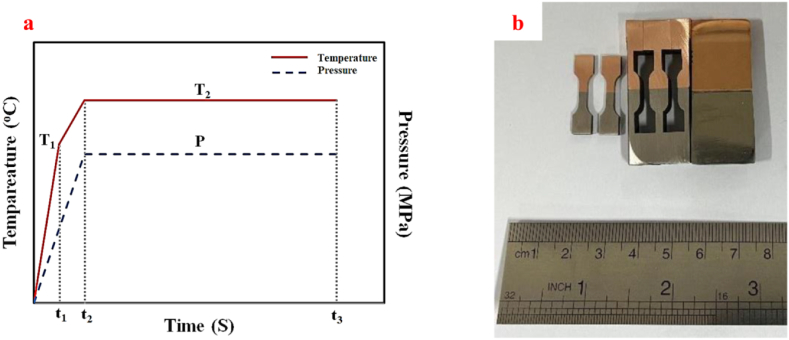
A schematic of heating and pressure application in SPW processes, and b) method of cutting the jointed samples.

After placing the samples inside the chamber and adjusting the temperature measurement region using a pyrometer, the chamber's lid was closed and the vacuum pump was initiated so that the air inside the chamber would be evacuated. After establishing the vacuum (around 1 mbar), SPW processes were done according to Fig. 3a. In all processes, first, within t_1_ = 180 s, the temperature was elevated up to T_1_ = 550 °C and at the time of t_2_ = 360 s, the temperature reached T_2_ = 650 °C. Once the temperature and pressure reached the desired value, the SPW process was done up to t_3_ = 2160 s. According to Fig. 3b, after the SPW process, for microscopic evaluation, the jointed samples were sectioned. Also, the tensile samples were prepared to evaluate the mechanical properties, according to the ASTM A370 standards.

To specify the diffusion affected zone (DAZ) via optical microscope, the cross-sectional area of the joints was electro-etched in 50 vol % H_3_PO_4_-50 vol % H_2_O solution with the voltage of 2 V. Also, the interface of the joints and fracture sections was evaluated via scanning electron microscopy (SEM) (JEOL7001F) equipped with energy dispersive spectroscopy (EDS). To assess the strength of the joints created via the SPW process, a tensile device was used. The tensile tests were conducted at 1 mm/min. The yield strength and joining strength were extracted from the engineering stress-strain curves.

### 2.1 Designing the temperature of the SPW process

Temperature, pressure, and time are the three main parameters of the SPW process. Since the SPW process is a diffusion solid-state joining process, the most important parameter in the SPW process is temperature. The SPW process is applied to join copper to steel through a metallurgical bond. The development of strong metallurgical bonds at the joining interface of solid-state processes would occur through the cross-diffusion of atoms. Thus, in the copper-steel joining process via SPW, the formation of a diffusion region between copper and steel depends on the effective diffusion of iron and copper. Considering the Arrhenius equation of diffusion, the most effective variable for atomic diffusion is the process temperature. Based on the iron-copper binary phase diagram, the extent of the solubility of copper in iron and iron in copper is close to zero at temperatures below 600 °C [41]. Thus, considering the iron-copper phase diagram, for designing the copper-steel SPW processes, temperatures above 600 °C should be considered.

Meanwhile, considering the initial assumptions and use of diffusion equations, the limits of the SPW process temperatures can be determined. Since the diffusion coefficient of copper in steel is lower than that of iron in copper [40], copper diffusion in steel was considered as the criterion for determining the desired temperature of the process. The equation of the diffusion coefficient of copper in iron has been presented by many researchers. The difference in the presented equations arises from different experimental conditions and the composition of materials [[41], [42], [43], [44], [45]]. In this research, the diffusion coefficient equation of Cu in Fe, as presented by Japan's Materials and Metals Institute, has been calculated with Equation (1) [42].

(1) $$D=0.47\;\exp\left(\frac{-244000}{RT}\right)\;{cm}^{2}s^{-1}$$

n the SPW process, in addition to temperature, the applied current also affects the diffusion of atoms at the interface due to electromigration and the development of a local temperature gradient. Based on the diffusion coefficient equation presented by Zhang et al. [40] (Equation (2)), electromigration and local temperature gradient are considered. Therefore, the effect of electric current on the diffusion of copper in steel was investigated.

(2) $$D=D_{0}\left(1+K_{1}I^{2}\pm K_{2}I\right),K_{1}=-\frac{R_{0}}{TK_{c}A_{c}}C_{i}\;\ln\;C_{i},K_{2}=\frac{R_{0}FZ_{i}^{*}}{TR}C_{i}$$

K_1_ refers to temperature gradient and K_2_ represents electromigration.

The applied current in the SPW Process affects the diffusion of atoms at the interface due to electromigration and the development of a local temperature gradient. Based on the diffusion coefficient equation presented by Zhang et al. [40] (Equation (2)), electromigration and local temperature gradient are considered for the SPW Process which has been verified by experimental results.

Also, according to equation (2), in the SPW process, the diffusion coefficient of atoms depends on temperature and electric current. K1 and K2 coefficients have been calculated according to the constants. According to these coefficients and equation (4) (dependence of electric current on temperature), finally, the diffusion coefficient depending on temperature is presented according to equation (5).

Considering the values of 3 × 10^−3^ Ω.cm for the contact resistance, 45 × 10^−2^ W/cm.k for the thermal conductivity coefficient of steel, 26.8 for the Faraday constant, 0.25 for the relative fraction, 3.14 cm^2^ for the area of the interface and 2.2 for an effective charge of copper, $K_{1}=\frac{7.38\times 10^{-4}}{T}$ and $K_{2}=\frac{5.29\times 10^{-3}}{T}$ were calculated. Thus, the equation of the diffusion coefficient of copper into steel (equation (3)), considering the electric current and assuming the aligned direction of copper diffusion and that of electric current, is:

(3) $$D=D_{0}\left(1+\frac{7.38\times 10^{-4}}{T}I^{2}+\frac{5.29\times 10^{-3}}{T}I\right),D_{0}=0.47\;\exp\left(\frac{-244000}{RT}\right)\;{cm}^{2}s^{-1}$$

n the SPW process, the electric current density is dependent on the process temperature and conditions (SPW device, materials and dimension of samples, and graphite separators). Based on the process conditions, the electric density equation has been obtained in terms of temperature (equation (4)):

(4) $$I=55\;\exp\left(0.0035T\right)\;A/{cm}^{2}$$

Therefore, the diffusion coefficient equation can be obtained as a temperature-dependent function (Equation (5)).

(5) $$D=0.47\;\exp\left(\frac{-244000}{RT}\right)\left(1+\frac{2.23\;\exp\left(0.007T\right)}{T}+\frac{0.29\;\exp\left(0.0035T\right)}{T}\right)$$

**Fig. 4:**
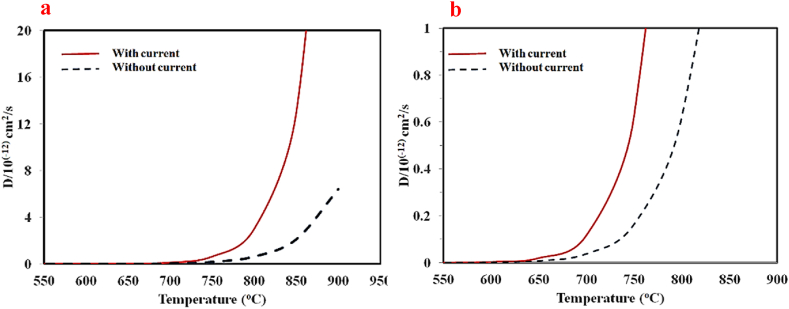
a) The diagram of the calculated diffusion coefficient of copper into iron under conditions of no electric current and presence of electric current aligned with the diffusion direction, and b) a magnified version of diagram (a) within the diffusion coefficient range 0-10^−12^ cm^2^/s.

Fig. 4a represents the diagram of the diffusion coefficient of Cu in Fe in terms of temperature without considering current and electric current aligned with the copper diffusion direction. By applying electric current along the copper diffusion, the extent of copper diffusion into steel would grow considerably. Fig. 4b shows the magnified range of diffusion coefficient 0-10^−12^ cm^2^/s. It can be predicted that in the process with the current applied along the copper diffusion, the ascending trend of copper diffusion coefficient into steel would begin from around 650 °C, and from about 725 °C for the current-free process. Thus, the copper-steel joining process temperature via the SPW process has been considered to be 650 °C. The samples were deployed such that they would be aligned with the electric current for copper diffusion into steel.

## 3 Results and discussion

**Fig. 5:**
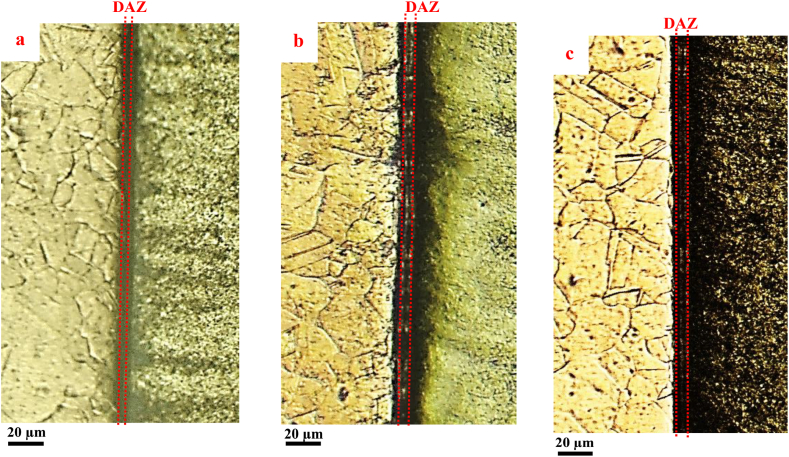
Optical microscopic images of the cross-section of joints under the SPW process at 650 °C for 30 min while applying pressures a) 20 MPa without mold, b) 20 MPa with mold, and c) 40 MPa with mold.

Fig. 5 demonstrates the optical microscopic images of a cross-section of copper-steel joints under the SPW process at 650 °C for 30 min and under the pressure of 20 MPa (without mold: Fig. 5a and with mold: Figs. 5b) and 40 MPa with mold (Fig. 5c). Through electro-etching of the cross-section close to the joint, diffusion-affected areas (DAZs) have been specified. The sample under the SPW process in the mold had more DAZs, as compared to the sample under the SPW process without mold. Since the extent of the solubility of iron in copper and copper in iron is very negligible, in copper-steel solid-state joining processes, forced mixing occurs due to the differences in the chemical concentration of atoms [31]. In the SPW process, the applied electric current and process temperature would lead to the diffusion of atoms from the jointed areas. Application of pressure, as well as volumetric and superficial diffusion, would result in a reduction of pores and an increase of the jointed areas. Thus, the atoms at the joining interface may be diffused and the areas close to the joint would also be affected by the diffusion of atoms. No considerable difference was observed in the DAZ of the joining interface of the samples under the SPW process and pressure of 20 MPa and 40 MPa.

**Fig. 6:**
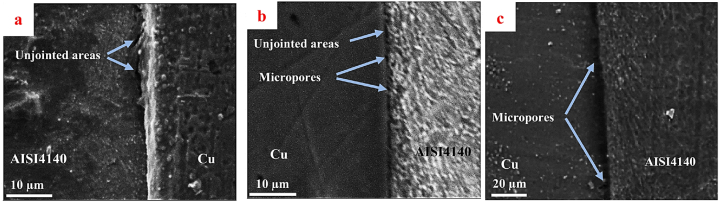
SEM images of the cross-section of the joints under the SPW process at 650 °C for 30 min, by applying the pressure of a) 20 MPa without mold, b) 20 MPa with mold, and c) 40 MPa with mold.

Fig. 6 presents the SEM images of the cross-section of the copper-steel joints under the SPW process at 650 °C for 30 min while applying the pressure of 20 MPa (with and without mold: Fig. 6a and b) and 40 MPa with mold (Fig. 6c). The unjointed areas at the interface of the sample under the SPW process without mold could be observed. In the sample under the SPW process with mold, the unjointed areas had shrunk, and some micropores could also be seen alongside these areas. According to the mechanism presented by Shen et al. [34], in the SPW process, pressure application together with spark discharge in the gap between the two samples would lead to the elimination of the unjointed surfaces between the two samples. Since temperature, time, and pressure were constant in both processes, there was another factor contributing to the reduction of the unjointed areas at the joint formed by the mold. As can also be seen in optical microscopy images (Fig. 5), at the joint generated in the mold, more DAZ was also formed, which resulted from the increase of the jointed areas and atomic diffusion paths. By comparing the processes done under the pressure of 20 MPa and 40 MPa, exertion of more pressure led to superior plastic deformation in the initial stages of the process, resulting in the faster removal of gaps and unjointed areas, as well as the creation of limited micropores at the copper-interface. The pressure applied in the SPW process causes the plastic deformation of the interface surfaces, leading to a shrunk gap and increased contact areas between two pieces [34]. The increase of the jointed areas between the two materials could result in the rise of the diffusion of atoms in the joint interface. In the process done with mold at 650 °C and pressure of 20 MPa, since the unjointed areas were partly removed and limited micropores were observed at the interface (Fig. 6b), and more interface surfaces were in contact with each other, by applying a higher pressure (40 MPa), the extent of the diffusion of atoms and DAZ did not change much.

**Fig. 7:**
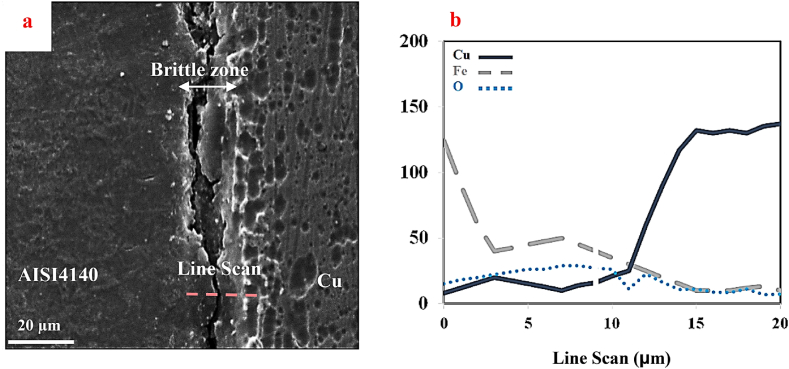
a) SEM image and b) iron, copper, and oxygen concentration profiles of the joint cross-section of the edge of the sample under the SPW process without mold at 650 °C for 30 min with a pressure of 20 MPa.

Fig. 7 indicates the SEM images (Fig. 7a) along with the concentration profile of iron, copper, and oxygen from the cross-section of the joint at areas close to the sample edge under the SPW process without mold (Fig. 7b). The unjointed areas could be observed throughout the entire interface. These areas were very brittle; upon preparation and etching of the joint's cross-section, parts of it were detached from the joint interface. This area contained a large percentage of iron, oxygen, and some copper.

**Fig. 8:**
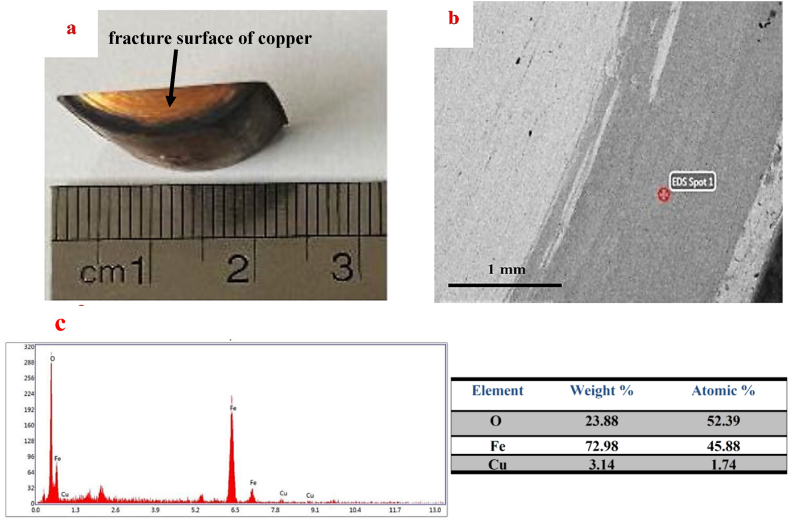
Image a: Actual, b) SEM image of the dark ring on the fracture surface of copper, and c) EDS analysis results on the dark fracture surface of copper in the SPW process without mold.

After the SPW process without metal mold, a dark ring was seen on the copper surface close to the external edge of the sample (Fig. 8a).

The fracture surface of copper is indicated by the arrow in Fig. 8a. Fig. 8a is the fracture surface of copper after the separation of the joint fabricated without mold process. As seen in Fig. 3b, the connected samples were cut for microscopic examination and hardness testing. In order to evaluate the fracture surface of the joint, one of the cut samples was separated by placing it in the tensile test fixture.

Fig. 8b shows the SEM image of the fractured area of copper. The fracture surface was relatively smooth with no plastic deformation, thus indicating the brittle fracture of the joint. By conducting EDS analysis on the dark surface (Fig. 8c), it could be found that this dark surface was iron oxide, which was formed without the mold process. This brittle oxide layer could also be seen at the cross-section of the joint edge (Fig. 7a). EDS results also revealed that 1.74 % of atomic copper had been diffused into the oxide layer. The results of the concentration profile of the cross-section of the joint edge (Fig. 7b) also confirmed the presence of copper in the oxide layer of the joint surface.

**Fig. 9:**
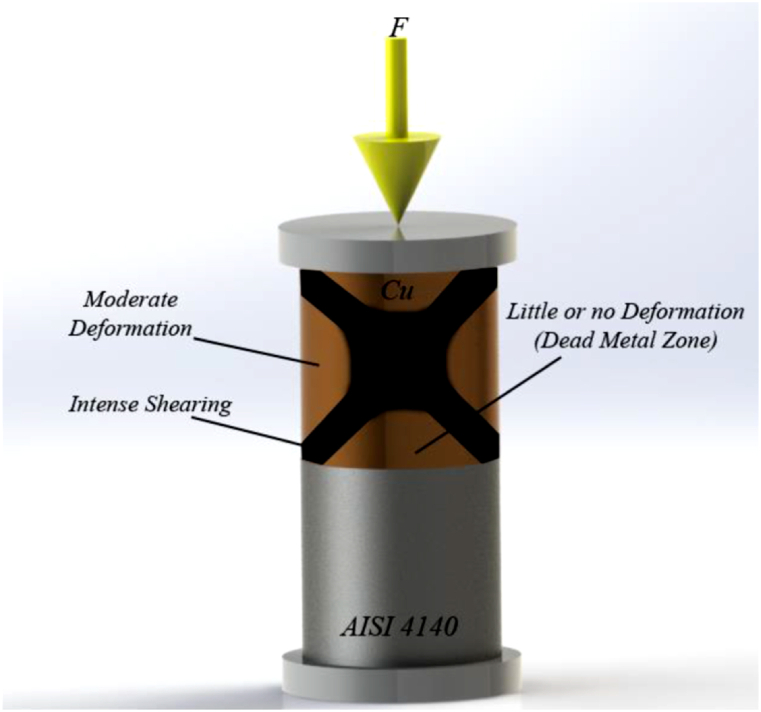
A schematic of the SPW process without mold and development of the dead zone.

Along the copper-steel joining process, though done under vacuum, the presence of oxygen at even trace amounts led to oxidation of the steel surface, especially at the edges of the sample. Under the SPW process without mold, oxidation of the surface of steel and joining of the steel to copper occurred simultaneously. As can be seen in Figs. 8c and 1.74 % atomic copper existed in the oxide layer, which resulted from copper diffusion into the oxide layer and the development of the joint between copper and the oxide layer on the steel surface. Another point that should be noted regarding the adhesion of the oxide layer to copper was the development of a dead zone in the center and severe deformation in the edge of samples along the SPW process without mold, as also noted by researchers considering the mold-free forging process of metals and alloys (Fig. 9) [43]. Thus, there was the probability of the formation of a dead zone in the central areas, as well as severe deformation areas, on the edges of the sample without mold the SPW process. Severe deformation in the edge of the sample contributed to the joining of the oxide layer to copper, and the presence of a dead zone in the central areas. The application of slight stress in these regions, caused copper plastic deformation not to occur properly at the copper-steel joint, and the unjointed areas could be seen at the copper-steel interface (Fig. 6a). Other reports were also survey properties of their product [[45], [46], [47], [48], [49], [50], [51], [52], [53], [54], [55], [56], [57], [58], [59], [60], [61]].

**Fig. 10:**
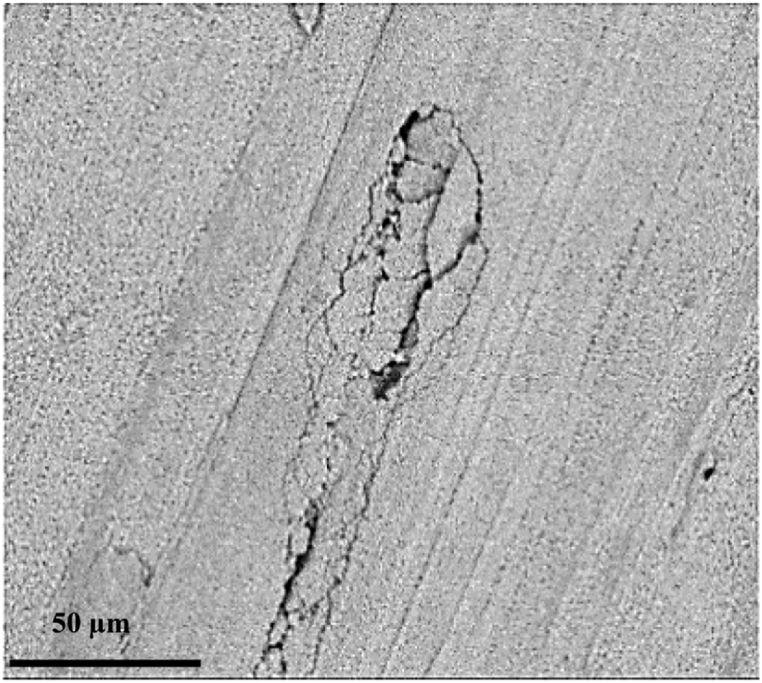
SEM image of crack development at joining interface in the dark oxide area in the SPW process without mold.

Fig. 10 shows an SEM image of the fracture surface of copper in the dark oxide area. Since steel oxide is brittle due to its porosity and has very low adhesion to the steel surface, in response to the stress applied at the joining site, a crack was formed, causing separation from the steel surface and remaining on the copper surface. As can be observed in Fig. 10, the cracks created at the oxidized region were due to the breakup of a region of the oxide layer of iron.

**Fig. 11:**
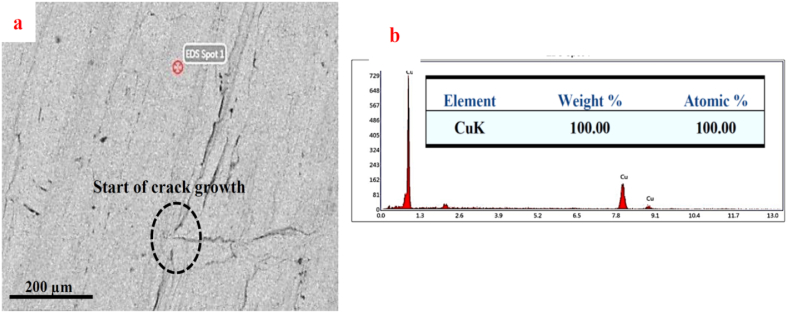
a) SEM image and b) EDS analysis of the fracture surface of copper in the areas farther away from the dark ring.

Fig. 11 indicates the SEM image (Fig. 11a) and the EDS analysis (Fig. 11b) of the copper fracture surface under the SPW process without mold in a region almost 1 mm away from the dark ring at the edge of the sample. The fracture surface of this region of the joint, like the dark area, was relatively smooth with no plastic deformation, thus indicating the brittle fracture of the joint. The cracks created in this region were different from those in the dark one. Based on SEM images, the cracks started from a region and propagated peripherally and radially towards the external edge of the sample until a fracture occurred. Considering the SEM image of the cross-section of the joint (Fig. 6a), the crack initiation area in the sample under the SPW process without mold could be the unjointed areas of the copper-steel interface. When moving away from the external surface towards the center of the joint, the extent of surface oxides of steel would diminish due to the lower access to the chamber environment, whereby higher strength would be developed in the joint. The crack movement towards the external edge of the sample was also due to the decreased joining strength towards the external edge. The results of EDS analysis (Fig. 11b) showed that iron diffusion into copper had not occurred or was very negligible considering the formation of an oxide layer on the steel surface and the presence of unjointed areas at the interface.

**Fig. 12:**
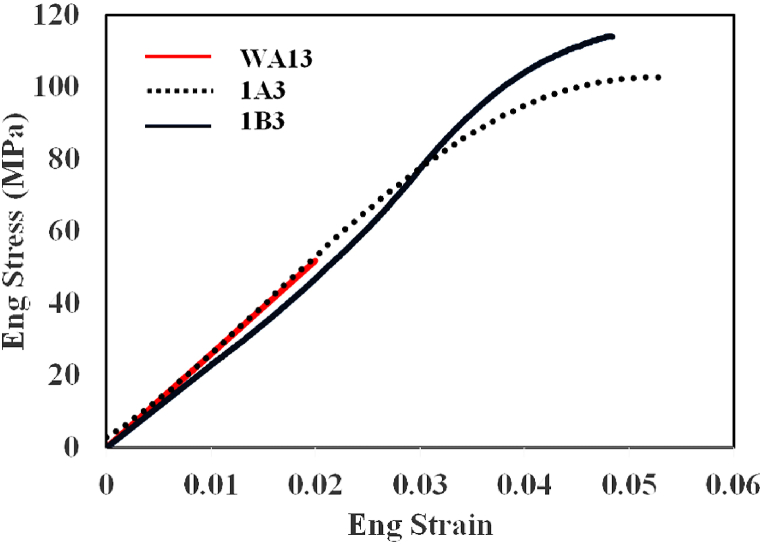
Engineering stress-strain curves of the joints created at 650 °C for 30 min, under the pressure of 20 MPa without (W1A3) and with mold (1A3), and 40 MPa with mold (1B3).

Fig. 12 indicates the engineering stress-strain curves of the copper-steel samples under the SPW process at 650 °C for 30 min by applying the pressure of 20 MPa (with and without mold) and 40 MPa with mold.

The true strength curve, which is not much different from the engineering strength, has been shown in the supporting information file (Fig. S1 and Table S1).

Fracture occurred in all three samples at the joining site. In the sample under the SPW process without mold, the joining at the elastic region was detached, and the samples under the SPW process with mold were taken apart in the plastic zone after yield stress. The yield strength is considered the starting point of plastic deformation. In the case of joints, the joining strength (ultimate tensile strength) is reported in this research. The copper's relatively smooth fracture surface with no plastic deformation (Fig. 10) also confirmed the brittle fracture in the sample under the SPW process without mold.

Table 2 reports the results of yield strength, joining strength and fracture strain of the joints created at 650 °C for 30 min under the pressure of 20 MPa (without and with mold) and 40 MPa with mold. The strength of the joints created without and with mold was obtained to be around 42 and 90 MPa, respectively. The results, thus, showed that through the SPW process in the mold, the tensile strength had grown by two times. Due to the increase in the strength of the joint created in the process with mold, the joint had been broken up after yield stress (88 MPa), with an elongation of around 1.8 %. The enhanced strength of the joint created in the mold could be attributed to the reduction of the unjointed areas and formation of limited micropores at the interface (Fig. 6), as well as greater diffusion of the atoms in the joint interface (Fig. 5). The joint formed at the pressure of 40 MPa had greater yield and tensile strength when compared to the joint created under the pressure of 20 MPa. The hardness of pure copper after the processes applied at 20 MPa and 40 MPa was obtained to be 49 HV_10_ and 59.5 HV_10_. The higher yield stress in the process under the pressure of 40 MPa indicated that the size of copper grains in this process had been smaller than in the process under the pressure of 20 MPa (for the joints created under the pressure of 20 and 40 MPa, 10–100 μm and 10–90 μm, respectively). The results obtained from pure copper hardness also confirmed this observation. Based on the SEM images of the cross-section of the samples under process with the pressure of 20 MPa (Figs. 6b) and 40 MPa (Fig. 6c), enhancement of the process strength with pressure elevation was also due to the removal of the unjointed areas and reduction of micropores at the joint interface. On the other hand, the joint formed under the pressure of 40 MPa had a lower failure strain due to the higher yield stress, as compared to the joint generated at the pressure 20 MPa.

**Table 2:** Yield strength, joining strength, and fracture strain of the joints created at 650 °C for 30 min under the pressure of 20 MPa without (w1A3) and with metal mold (1A3), and 40 MPa with mold (1B3).

Sample Code	Yield strength (MPa)	joining strength (MPa)	Failure Strain
w1A3	–	42 ± 8	0
1A3	88 ± 2	90 ± 11	0.018 ± 0.005
1B3	99 ± 2	106 ± 9	0.012 ± 0.004

**Fig. 13:**
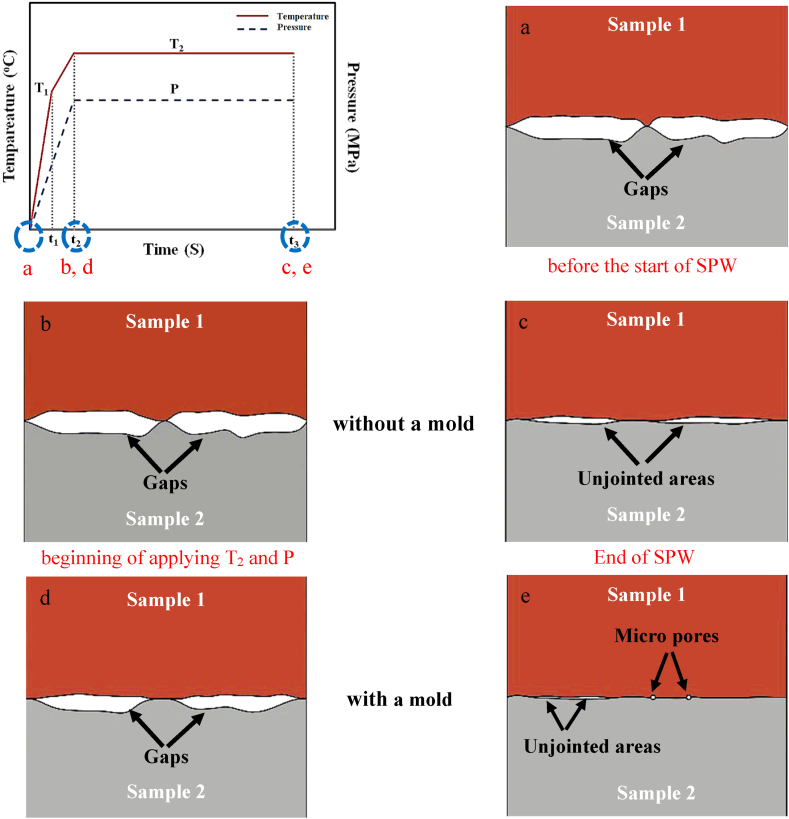
Schematic of the joint interface a) before the start of the SPW process, b) at the beginning of applying the temperature (T2) and pressure (P) in the SPW process without a mold were applied, c) at the end of the SPW process without a mold, d) at the beginning of applying the temperature (T2) and pressure (P) in the SPW process with a mold, and e) at the end of the SPW process without a mold.

According to the obtained results, a mechanism for the SPW process without and with a mold in the joint interface has been presented. As shown in Fig. 13a, at the beginning of the processes without and with a mold, there were limited contact points and gaps at the interface of the two samples. By applying pressure and electric current to the samples to reach the temperature and pressure of the process (T_2_ and P), according to the graph of Fig. 3a, partial plastic deformation occurred at the interface of the samples under the process without a mold due to the presence of the dead zone. Thus, the interface of the samples contained limited contact points and gaps as at the beginning of the process (Fig. 13b). As a result of current passing and spark discharge in the gaps of the interface during the SPW process without a mold, the diffusion of atoms took place through the limited connection areas, and the gaps of the interface disappeared and became unjointed areas (Figure 13c. In the SPW process with a mold, by applying pressure and electric current to the samples to reach the temperature and pressure of the process (P and T2), at the interface of the samples, the plastic deformation of the copper was created due to the applied pressure, P, causing the interface of the samples to contain more contact points and fewer gaps than that at the beginning of the process (Fig. 13d). As a result of current passing and spark discharge in the interface gaps during the SPW process, diffusion of atoms through the connection areas, which were more than that by the process without a mold, took place; at the end of the process, the interface gaps disappeared. Thus, the joint interface included limited unjointed areas and micropores (Fig. 13e).

## 4 Conclusion

Based on the SPW processes done with and without mold, at 650 °C for 30 min, under the pressure of 20 and 40 MPa, according to microstructures evaluations and characterization of the mechanical properties of the joints, the results can be summarized as follows:

- The SPW process without mold at the starting temperature of the ascending trend of the diffusion coefficient of atoms in the interface joint led to the oxidation of the steel surface, especially at the joint edge, and the development of a dead zone at the joint interface. Thus, little plastic deformation could occur in the dead zone and severe deformation might occur at the edges of the joint interface, leading to the joining of the iron oxide layer to the copper at the edges and the presence of the unjointed areas at the interface joint.
- As a result of the presence of the unjointed areas in the joint interface of the SPW process without mold, brittle fractures in the elastic area occurred in the tensile test.
- By performing the SPW process with mold at the starting temperature of the ascending trend of the diffusion coefficient of atoms in the interface joint due to the plastic deformation of the joint interface caused by the applied pressure, reduction of unjointed areas in the interface, as well as an increase in DAZ, as compared to the mold-free process, the joining strength was increased from 42 to 90 MPa.
- As a result of the presence of limited unjointed areas and micropores in the joint interface of the SPW process with mold, the fracture occurred after the yield stress and in the plastic region.
- An increase of the SPW process pressure from 20 MPa to 40 MPa, despite the removal of limited unjointed areas and the presence of micropores, did not have a perceptible effect on increasing DAZ and joining strength.

## Ethical approval

All Authors confirm that we have reviewed journal guidelines for Ethics in Publishing as well as Heliyon's Ethics and Editorial Policies.

## Data availability statement

Data is available on the request of editor.

## CRediT authorship contribution statement

**Mehdi Naderi:** Conceptualization, Data curation, Formal analysis, Investigation, Methodology, Project administration, Writing – original draft. **Mohammad Reza Toroghinejad:** Formal analysis, Project administration, Resources, Supervision, Validation, Writing – review & editing. **Ahmad Kermanpur:** Formal analysis, Project administration, Supervision, Validation.

## Declaration of competing interest

The authors declare that they have no known competing financial interests or personal relationships that could have appeared to influence the work reported in this paper.
