# Supplementary material for: Spark plasma welding joining of copper- AISI4140 steel: Microstructures and mechanical properties
Source: Heliyon. 2023 Oct 23;9(11):e21364. doi: 10.1016/j.heliyon.2023.e21364 (PMC10641177; doi:10.1016/j.heliyon.2023.e21364)
Supplement: Multimedia component 1 [file mmc1.docx]

**
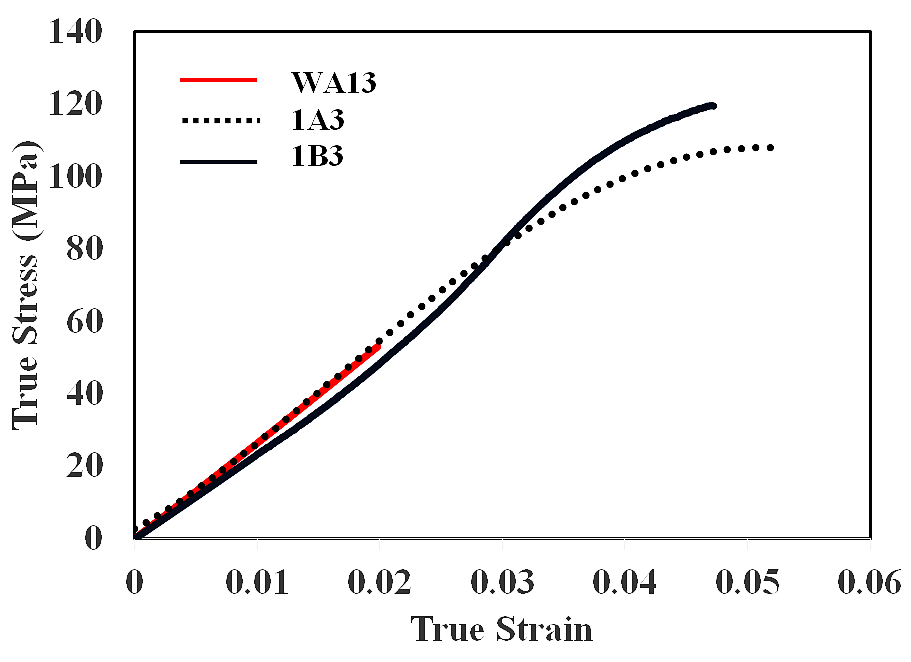
Supporting information file**

Fig. S1. True stress-strain curves of the joints created at 650˚C for 30 min, under the pressure of 20MPa without (W1A3) and with mold (1A3), and 40MPa with mold (1B3).

Table S1- Yield strength, True joining strength, and True fracture strain of the joints created at 650 ^o^C for 30 min under the pressure of 20 MPa without (w1A3) and with metal mold (1A3), and 40 MPa with mold (1B3).

| **Failure Strain** | **joining strength)MPa(** | **Yield strength (MPa)** | **Sample Code** |
| --- | --- | --- | --- |
| 0 | 44±8 | - | w1A3 |
| 0.017±0.005 | 96±12 | 91±2 | 1A3 |
| 0.011±0.005 | 111±8 | 103±2 | 1B3 |
